# Identifying research priorities for public health research to address health inequalities: use of Delphi-like survey methods

**DOI:** 10.1186/s12961-017-0252-2

**Published:** 2017-10-09

**Authors:** S. Turner, E. Ollerhead, A. Cook

**Affiliations:** 1 National Institute for Health Research, Evaluation, Trials and Studies Coordinating Centre (NETSCC), University of Southampton, Alpha House, Enterprise Road, Southampton, SO16 7NS United Kingdom; 2 Wessex Institute, University of Southampton, Alpha House, Enterprise Road, Southampton, SO16 7NS United Kingdom; 3 Southampton University Hospitals Trust, Tremona Road, Southampton, United Kingdom

**Keywords:** Research need, Research priority, Research funding, Delphi-like methods, Health inequalities, Complex systems

## Abstract

**Background:**

In the funding of health research and public health research it is vital that research questions posed are important and that funded research meets a research need or a gap in evidence. Many methods are used in the identification of research priorities, however, these can be resource intensive, costly and logistically challenging. Identifying such research priorities can be particularly challenging for complex public health problems as there is a need to consult a number of experts across disciplines and with a range of expertise. This study investigated the use of Delphi-like survey methods in identifying important research priorities relating to health inequalities and framing tractable research questions for topic areas identified.

**Methods:**

The study was conducted in two phases, both using Delphi-like survey methods. Firstly, public health professionals with an interest in health inequalities were asked to identify research priorities. Secondly academic researchers were asked to frame tractable research questions relating to the priorities identified. These research priorities identified using Delphi-like survey methods were subsequently compared to those identified using different methods.

**Results:**

A total of 52 public health professionals and 21 academics across the United Kingdom agreed to take part. The response rates were high, from public health professionals across three survey rounds (69%, 50% and 40%) and from academics across one round (52%), indicating that participants were receptive to the method and motivated to respond. The themes identified as encompassing the most important research priorities were mental health, healthy environment and health behaviours. Within these themes, the topic areas that emerged most strongly included community interventions for prevention of mental health problems and the food and alcohol environment. Some responses received from academic researchers were (as requested) in the form of tractable research questions, whereas others contributed further potential topic areas instead.

**Conclusions:**

Delphi-like survey methods are practical and productive as a means of obtaining opinions from a wide number of relevant experts identifying potential priority topic areas for research; however, this method is less appropriate for framing tractable research questions.

**Electronic supplementary material:**

The online version of this article (doi:10.1186/s12961-017-0252-2) contains supplementary material, which is available to authorized users.

## Background

In the funding of health research and public health research, it is essential that the research questions addressed are important to patients and the health of the public, and that funded research meets a research need or a gap in evidence [1, 2]. It is also vital that there is a connection between policy, practice and research [3, 4]. The identification of research needs is a complex task, with literature describing a wide range of methods for identifying research topics, but little information on how well the various methods work [5, 6]. The National Institute for Health Research (NIHR) Public Health Research (PHR) programme funds research via two funding streams. In the researcher-led funding stream, the researchers submit applications for funding on topics of their choosing. In the commissioned work stream, the NIHR PHR programme aims to identify topics of high public health importance in order to commission research to provide evidence where it is most needed. However, for some areas of practice, the evidence is sparse and the issues wide and complex, and it is not always clear where the gaps are, which are the highest priorities for research, or how the issues may be addressed.

The remit of the PHR programme is very broad and members of the prioritisation board (Programme Advisory Board (PAB) – the body that decides on the priority areas for the programme to pursue) cannot possibly cover all the relevant areas of specialist expertise. What is required is expert input in identifying priorities to augment the expertise of the membership on the board and to help the programme focus on the issues where research is most needed. What is also needed is help with framing tractable research questions. Identifying an important public health problem is only the first stage in commissioning research. There is an important stage in the research process where an identified public health ‘problem’ needs to be ‘framed’ as a tractable research question with suitable research designs considered [7]. While those working in the field of public health are well placed to identify the ‘problems’, and are often equipped to critically appraise research, they are not usually so well versed in research design, which is more usually the domain of academic researchers. However, the academic researchers may not be so familiar with the ‘problems’ and priorities for which research evidence is required [4].

The NIHR PHR programme requires methods to solicit opinions from those working in the field of public health with the necessary knowledge, experience and expertise as to what its research priorities should be. The NIHR PHR programme currently uses a wide variety of methods for topic identification and priority setting, including workshops, literature scanning, suggestions submitted through a website, and face-to-face engagement activity. Some of these methods are time consuming, labour intensive and may lack informed expert opinion; additionally, the practical challenges of getting a group of experts in the same place at the same time for a workshop or face-to-face priority setting activity is considerable, as well as costly. Thus, the programme requires an efficient and effective method of tapping into timely specialist expertise from a wide range of experts on particular topics, and which is not resource intensive either with regards to human resources or expenditure on hiring premises for boards/consultative events. In short, a way of consulting widely, focusing our efforts and producing ‘researchable’ topics addressing the most important needs of policy and practice customers.

Delphi surveys have been previously used for topic identification [8–17]. There are many advantages in using this type of method, including that many people can be consulted without the constraints of having a face-to-face meeting (busy people are not always available for group activities), it allows access into specialist expertise from a wide range of experts, on particular topics and without investing considerable amounts of human and cost resources, and participants can contribute at their own convenience; further, this method also allows for iteration around the topic in question.

There are few studies comparing methods for topic identification. Previous work by Chase et al. [18] investigated the relative merits of different sources used to identify potential research questions. They found that the largest numbers of suggested topics came from widespread consultation with healthcare commissioners, providers and consumers; however, the success rate from this source in terms of research commissioned was low. A Delphi approach was used by Bambra et al. [19] for similar purposes in developing policy. Finally, other funding organisations and professional groups have also used modified forms of this approach for setting research priorities [8–17, 20–23].

A current research priority area for the NIHR PHR programme is that of health inequalities, which is also a United Kingdom priority in policy and practice. This is an example of a very wide ranging and complex subject that requires a wide range of expert knowledge. It is a complex area where the programme has encountered difficulties refining topics in the past. The aim of the first phase of this study was to investigate the application of Delphi-like survey methods in identifying important topics for public health research that address health inequalities. The aim of the second phase of the study was to investigate the use of Delphi-like survey methods in framing tractable research questions for the problems/priorities identified.

## Methods

### Overview

The study was conducted in two phases. Phase 1 consisted of three Delphi-like survey rounds (Additional file 1) sent to public health professionals and those working in the field of public health in the United Kingdom, with an interest in health inequalities. Participants were asked about questions/problems in health inequalities in the United Kingdom where evidence would be helpful and how these problems might be tackled.

In Phase 2, a Delphi-like survey (Additional file 1) was sent to a group of research-active methodologists/academics, identified as experts in the areas of the topics identified in Phase 1. Participants in this phase were asked to frame tractable research questions for these two topic areas, and to alert us to relevant methodological issues that should be mentioned in the commissioning brief.

Ethics approval was sought and obtained from the University of Southampton Ethics and Research Governance Online, submission number 8990.

### Phase 1

#### Selection of participants

The selected participants were public health professionals and those working in the field of public health in the United Kingdom, with an interest in health inequalities. We sought to involve people with a role in public health service delivery. Potential participants were recruited through various sources, including Contact, Help, Advice and Information Network (CHAIN) [24], contacts made through conferences and meetings, Association of Directors of Adult Social Services [25] (ADASS; list of members available on internet), Directors of Public Health identified on the internet, and through the delegate list of the Local Government Association Annual Public Health conference held in 2015. The numbers of people contacted from the various sources are shown in Table 1. Potential participants were sent an e-mail explaining the rationale and aims of the study, why they had been selected to receive an invitation to participate, and what would be involved should they agree to take part.

**Table 1:** Sources for recruitment of participants and response rates for Phase 1

	Numbers contacted	Positive replies	Percentage response	Number who completed Round 1 survey	Percentage of responders who completed Round 1 survey	Number who completed Round 2 survey	Percentage of responders who completed survey Round 2	Number who completed Round 3 survey	Percentage of responders who completed survey Round 3
Through CHAIN	41	29	71%	22	76%	19	66%	13	45%
Snowballing from CHAIN	6	3	50%	3	100%	2	67%	2	67%
Personal contacts	4	4	100%	3	75%	2	50%	2	50%
ADASS	59	10	17%	5	50%	1	10%	1	10%
Snowballing from ADASS	2	2	100%	0	0%	0	0%	1	50%
Directors of PH/LGA delegate list	25	4	16%	3	75%	2	50%	2	50%
TOTAL	137	52	38%	36	69%	26	50%	21	40%
Overall response rate from persons initially contacted					26%		19%		15%

*ADASS* Association of Directors of Adult Social Services, *CHAIN* Contact, Help, Advice and Information Network, *LGA*, Local Government Association, *PH* public health

#### Delphi survey round 1

Those who agreed to participate were sent the first round of the Delphi survey via Survey Monkey, in which they were asked “In your opinion, what are the most important questions/problems in health inequalities in the United Kingdom where evidence would be helpful? What are your reasons for selecting this/these question(s)/problem(s)?” They were asked to give a maximum of three responses in free text. Participants were also asked about their job title, the organisation they worked for, population numbers served and the geographical location of their organisation within the United Kingdom (Table 2). As participants were self-selected as having an interest in ‘health inequalities’, we did not provide a definition of the term but expected that the definitions used by the National Institute for Health and Care Excellence [26] or the Department of Health [27] would be widely known. The responses were analysed and the themes most frequently mentioned by respondents identified.

**Table 2:** Participant characteristics

	Number of participants		
Geographical location	Round 1	Round 2	Round 3
London	8	7	6
Midlands and East of England	9	6	3
South of England	2	3	0
North of England	12	8	7
Northern Ireland	0	0	0
Scotland	3	2	2
Wales	0	0	1
All regions	1	0	0
Not answered	1	0	2
Total	36	26	21
Organisation			
Lower tier	2	0	1
Upper tier	5	1	3
Unitary	16	13	8
Other	13	9	7
Not answered	0	3	2
Total	36	26	21
Population served (in thousands)			
< 100	2	4	5
100–500	19	12	10
> 500	12	10	3
Not answered	3	0	3
Total	36	26	21

#### Delphi survey round 2

In Round 2 of the survey, the top three themes identified by the group in Round 1 were fed back to the group, and participants were asked “In which areas of your practice related to …1/2/3… do you feel that you need new information/evidence?” and “Would your practice be likely to change if there was new evidence?” The responses from Round 2 were analysed and the topic areas mentioned most frequently were identified.

#### Delphi survey round 3

Three ‘top’ topic areas (those mentioned most frequently) were selected from the responses to Round 2, and fed back to the group. The aim of Round 3 was to further refine the ideas put forward by the group in the previous round and to ask their opinions about how these ‘top three’ problem topic areas may be tackled. The main topic areas were listed in the survey, and participants were asked: “Which interventions (things that can be done) would you like to see researched and in which population groups would you like to see this research done?” The responses from Round 3 were analysed and the areas requiring intervention and the population groups most frequently mentioned were identified.

#### Statistical analysis

We were interested in whether the source of participants affected their contribution to the subsequent work. Our prior hypothesis was that people sourced through CHAIN would be more likely to participate than those contacted through other sources, as they have passed through a prior screening process organised by CHAIN and hence had shown some interest, whereas the other groups were effectively cold-called.

We investigated whether there was a difference between the groups in the proportion of people approached who agreed to participate and, of those who agreed to participate, whether there was a relationship between the source of the individual and whether they participated in each of the three rounds. Given the planned significance tests, we applied Bonferroni’s correction, redefining the limit of significance as 0.0125 rather than 0.05. All statistical analyses were undertaken using R version 3.4.0 [28].

### Phase 2

#### Selection of participants and survey

Participants for Phase 2 were a group of research-active methodologists/academics, who were experts in the areas of the topics identified in Phase 1. Potential participants were identified through CHAIN and searching the internet for experts in the field (from staff lists of relevant research groups and authors of relevant publications). Letters of invitation were sent to potential participants asking them to take part in a survey. The survey was sent to those who agreed to take part and participants were asked:

“As explained in our letter inviting you to participate in this study, The NIHR Public Health Research Programme is interested in advertising for research addressing health inequalities. Our participants in Phase 1 of this study have identified the following ‘topic areas’ as high priorities for research: ‘Community interventions for prevention of mental health problems’ and ‘Food and alcohol environment’. They have told us that there is a need for research evidence to inform practice. We would like to draw on your expertise to help us frame tractable research questions for these two topic areas, and to alert us to relevant methodological issues which should be mentioned in the commissioning brief (the call advertising for research).”

Responses to Phase 2 were collated in lists within these topic areas.

#### Evaluation

Lists of responses to questions posed in Phase 2 were compiled for the three topic areas of (1) food environment, (2) alcohol environment and (3) community interventions for the prevention of mental health problems. Corresponding lists of research topics from these topic areas, identified by methods used previously (topic identification workshops at PAB, suggestions submitted through NIHR website, literature scanning and research recommendations from completed NIHR studies), were compiled for comparison. Pairs of lists were constructed for each topic where, for each area, one list was that compiled using Delphi-like methods and the other was that compiled using other methods. Within each pair of lists, each assessor was presented with the lists in random order to avoid ordering bias, with the first list they were presented with labelled A, and the second B.

Hard copies of the lists of topic areas for comparison were taken to a meeting of the prioritisation board for the NIHR PHR programme, following the usual voting process by which topics are prioritised for research funding. The function of the board is to identify areas of research where there is greatest need for research evidence. Members of the board were asked “For each pair of lists please choose which list you would prefer to prioritise, A or B?” Members were asked to return the lists in a pre-paid envelope and lists were sent to non-attenders by e-mail.

## Results

### Phase 1

#### Selection of participants

An invitation to participate was sent to 137 potential participants; the different sources used to identify the potential participants are shown in Table 1. Of the 137 people contacted, 52 replied and agreed to participate (38%), which may be considered a high response rate for an external survey [29]. Highest response rates were observed for those contacted via personal contacts, snowballing from ADASS, and CHAIN. This is unsurprising as these participants were self-selected; other groups were contacted through ‘cold calling’ by e-mail.

Round 1 of the survey was sent to the 52 people who agreed to participate, all of whom had identified themselves as public health professionals; 36 people completed and returned the survey (69% response rate). Again, the highest response rates were from participants recruited via CHAIN and through personal contacts (Table 1).

The response rates of participants from different sources for all three rounds are also shown in Table 1. The participant characteristics are shown in Table 2.

#### Delphi survey round 1

The themes identified as encompassing the most important research priorities were identified as those with the most responses. The highest numbers of responses were related to mental health. Specific topic areas within this theme mentioned by respondents included prevention of serious mental health problems; building resilience and self-esteem; the mental health of specific groups, including young people and school children, women, members of minority ethnic communities and older people; dementia; and the link between mental health and the social determinants of health.

The other themes with high numbers of responses centred on healthy environment and health behaviours, and some of the topic areas mentioned were inter-linked. Topic areas mentioned by respondents included alcohol consumption; overweight, obesity and healthy eating; inactivity; smoking; the impact of providing and maintaining good quality green and natural spaces and access to those spaces; the built environment, including housing and areas of high deprivation; transport; and issues relating to rural communities.

As part of the Round 1 survey, participants were asked to state their job title. Upon analysis of the responses to Round 1, it became apparent that four of the responses were from academics (one each for Professor of Public Health, Associate Professor, Senior Lecturer and Senior Research Fellow). The intention had been to contact academics in Phase 2, however, their responses to Round 1 were included since they were available.

#### Delphi survey round 2

Round 2 of the survey was sent to all 52 participants, 26 replies were received (50% response rate). In this round, participants had been asked to identify which topic areas within the main themes they thought most required research evidence. Responses were collated by theme (mental health, healthy environment and health behaviours).

Every respondent commented on mental health, which emerged as the strongest theme (Table 3). There was an emphasis on resilience, self-esteem, prevention, early diagnosis, social isolation and mental health in minority ethnic communities.

**Table 3:** Responses to Round 2

Theme	Answered	Skipped
Mental health	26	0
Healthy environment	24	2
Health behaviours	24	2

Within the theme of healthy environment, the topic areas that emerged strongly included the food environment, with particular focus on obesity, fast food outlets and planning. Other topics included green space, active transport and housing quality.

Within the theme of health behaviours, topic areas which emerged most strongly included obesity, and multiple health behaviours including alcohol use and the alcohol environment. Other topic areas included physical activity.

#### Delphi survey round 3

The same three themes were taken forward for Round 3; in this round, participants had been asked to provide their views about how these issues might be tackled. Round 3 of the survey was sent to all 52 participants, 21 replies were received (40% response rate).

Some of the respondents did not answer the questions as posed, but topic areas in responses could be identified. There were too many topic areas identified to be able to carry them all forward within the scope of this project; therefore, only two were selected to take forward into Phase 2, namely community interventions for prevention of mental health problems and the food and alcohol environment. The reasons for selecting these themes were that they were high on the list from respondents, they were topic areas within which we had previously tried to identify topics through other methods and therefore a comparator was available for evaluation, and these were areas in which the NIHR portfolio was lacking and we would therefore want to commission research.

The number of survey rounds completed by participants, and the sources through which they were identified, are shown in Table 4.

**Table 4:** Rounds completed by participants in Phase 1

	Completed 3 rounds	Completed 2 rounds	Completed 1 round	Completed 0 rounds	Total responses
Through CHAIN	11	6	9	3	29
Snowballing from CHAIN	2	0	1	0	3
Personal contacts	1	1	2	0	4
ADASS	0	1	5	4	10
Snowballing from ADASS	0	0	1	1	2
Directors of PH/LGA delegate list	1	2	0	1	4
Totals per round	15	10	18	9	52

*ADASS* Association of Directors of Adult Social Services, *CHAIN* Contact, Help, Advice and Information Network, *LGA*, Local Government Association, *PH* public health

#### Statistical analysis

We tested a null hypothesis of no difference between the source of participants and their agreement to take part in the Delphi-like survey. The *P* value for agreeing to participate in the Delphi-like survey was 0.00000001. The *P* values for participating in each of the three rounds were 0.14, 0.04 and 0.37. While the Round 2 test would conventionally be significant, due to Bonferroni’s correction, it was not. We therefore concluded that the source of the participant influenced their initial agreement to take part in the process, but once someone had agreed to participate, the source had no impact on whether they completed the requested work.

We then considered whether the source of an expert might impact how many rounds they contributed to. Given that the underlying data cannot be demonstrated to be distributed normally, we approached this using the Kruskal–Wallis test, using the null hypothesis that the source would have no impact on participation. The *P* value for this was 0.03. Further investigation using Dunn’s test with the Bonferroni correction for multiplicity showed the only paired relationship with a significant effect at the 5% level was that between CHAIN and the ADASS (*P* = 0.023 after Bonferroni correction). We conclude that participants sourced through CHAIN were more likely to contribute to multiple rounds than those sourced through the ADASS. As mentioned above, it would seem that this is because participants sourced through CHAIN were actively engaged with the process before being invited to participate, whereas the other groups were effectively cold called. No relationship between geography, organisation or population served was found, with Fisher’s exact test giving *P* values of 0.83, 0.41 and 0.15, respectively.

### Phase 2

#### Selection of participants and survey

A total of 47 participants were identified through CHAIN (n = 8) and the internet (n = 39) (Table 5). Of those invited to participate, 21 agreed and 11 responses to the survey were subsequently received (52% response rate). Of the 11 participants, eight were professors, one was a clinical senior lecturer, one a lecturer and one a research fellow. They were all from universities within the United Kingdom (four in the North of England, three in the South of England, and one each in the Midlands, London, Scotland and Wales).

**Table 5:** Selection of participants and survey response

	n or %
Number of participants invited	47
Replied Yes	21
Replied No	3
No response	23
Surveys sent	21
Surveys completed (response rate 52%)	11
Overall response rate from persons initially contacted	23%

Participants had been asked to help by forming tractable research questions for these topic areas and alerting us to any relevant methodological issues that we should consider. Some responses received were in the form of research questions, others contributed further potential topic areas within the scope of the suggested topics areas. Several of the respondents returned more than one suggestion. Responses to the survey were collated into lists within topic areas.

#### Evaluation

Eighteen members of PAB were asked for their opinions on the list of topic areas, 12 responses were received (response rate 66%). The stated preferences for the two lists are shown in Table 6.

**Table 6:** Stated preferences of PAB members for the two lists

	Delphi methodology		Other methods		No preference	Total responses
	Strong	Weak	Strong	Weak		
Food environment	0	0	7	3	2	12
Alcohol environment	2	4	3	2	1	12
Mental health	1	2	3	4	2	12
Total	9		22		5	

For ‘Alcohol environment’, in total, six members voted for the list compiled using Delphi-like survey methods and five voted for the list compiled using ‘other methods’ (two stating a strong preference, four a weak preference). The group who chose the Delphi list did so because it was judged to be more ‘upstream’, encompassing the whole system and broad. The reasons for choosing ‘other methods’ were mixed, with reasons including being more grounded and realistic.

For ‘Food environment’, the group preferred the list compiled using ‘other methods’; this list was judged to be more ‘upstream’ and the list compiled using Delphi-like survey methods was considered more ‘downstream’. The ‘other methods’ list was also described as more grounded and implementable.

For ‘Mental health’, in total, there were three votes for the list compiled using Delphi-like survey methods and seven for ‘other methods’. This list was preferred as it was judged to be clearer, more holistic, implementable and likely to generate results.

Additional file 1 shows the lists of topic suggestions; for clarity, in this document, the lists have been labelled as either ‘Delphi-like survey methods’ or ‘Other methods’, although PAB members were blinded as to the nature of each list and, within each pair, the lists were presented in a random order.

## Discussion

The findings of this study demonstrate that Delphi-like survey methods are practical and productive in identifying topic areas, within a complex system, where research evidence is needed. This method allowed a diverse range of experts in relevant fields of expertise to be consulted and offered a more economical approach than a face-to-face meeting. Difficulties associated with face-to-face meetings include the practicalities and costs of providing a venue large enough for such a meeting, and the feasibility of arranging a time convenient to all. The problems associated with arranging such meetings involving multiple stakeholders across the United Kingdom are often alleviated by using Delphi-like survey methods. This allows participants to respond at a time convenient to them, after taking the time they require to consider their responses, and no notice period in advance of a meeting is required. Some resource is required, however, for the organisation of the survey.

Nevertheless, this method was less productive in terms of obtaining tractable research questions. The participants in Phase 1 were instrumental in identifying the ‘problems’ where research evidence was needed, but when these ‘problems’ relating to complex systems were then offered to academic researchers to formulate tractable research questions, in some cases, respondents did not do this but instead suggested further potential topics for research. Where they did formulate research questions, the researchers tended to suggest questions which they perceived as researchable or ‘do-able’ within the paradigm of the complex system. When referred back to the body used to prioritising on the basis of public health importance, however, their preference was for topics or questions which they perceived as ‘upstream’ and whole system, but which could be extremely challenging to research. This highlights the potential disconnect that has been noted previously between practice and research [4]. While it is a legitimate aim of a research funder to identify evidence gaps and thus commission research based on the needs of those working in policy and practice, it must be feasible to deliver the research that is commissioned.

As stated previously, it is vitally important that information generated through robust research exists to inform public health policy and practice. Public health professionals need research evidence to help them with their day-to-day practice, interventions need to be developed and assessed on effectiveness and cost effectiveness, and there is also a need for research to inform commissioning decisions [4, 30]. However, there are difficulties in connecting academic research and practice and policy [4]. The process has been described by Jansen et al. [4], who characterised the steps in the process of policymaking. The first step is problem recognition, followed by an analysis of the problem and the formulation of an approach to solve it, which is step 2. Step 3 involves the initiation of implementation and, finally, step 4, when the effects are interpreted and evaluated [4]. At each stage, there are risks of disconnection. It would be helpful if a more effective dialogue and connection between these groups could be established.

The strengths of this study were that it generated many potential topic areas where research was needed, we were able to reach a range of experts in the relevant fields to ask their views and response rates were good, indicating that participants were receptive to the method and motivated to respond. This study also highlighted the value of a commissioned work stream focused on research need. However, this method was not so useful for generating tractable research questions.

A weakness of the evaluation stage was that some of the topic areas included in the lists had been generated at workshops attended by members of the PAB, so although there is planned membership turnover, some of the board members choosing between the lists may have seen some of the topic areas previously. However, the main function of this board is to prioritise topic areas where there is the greatest need for research evidence, so it was well placed to make this judgement. A small number of academics were included in Phase 1, and this did not become apparent until the data was analysed in detail. However, this represented only a small percentage of respondents.

This method proved useful in identifying research need for the themes included in this study, but not so useful in framing tractable research questions. Delphi-like survey methods could be considered as one of a range of approaches that may be used to identify research priorities. Further work investigating the use of this type of method for other complex health research problems, including interdisciplinary topic areas, is needed. An investigation using different methodologies for different topics or types of questions would also be informative.

## Conclusions

This study has shown that Delphi-like survey methods are practical and productive as a means of obtaining opinions about evidence gaps from a wide number of relevant experts and identifying potential priority topic areas for research. However, the method is not so helpful in eliciting support from experts in framing tractable research questions.

## Additional file

Additional file 1: Surveys - Rounds 1, 2 and 3. (PDF 275 kb)

## Abbreviations

NIHR: National Institute for Health Research
PAB: Programme Advisory Board
PHR: Public Health Research
