# Supplementary material for: Identifying research priorities for public health research to address health inequalities: use of Delphi-like survey methods
Source: Health Res Policy Syst. 2017 Oct 9;15:87. doi: 10.1186/s12961-017-0252-2 (PMC5632826; doi:10.1186/s12961-017-0252-2)
Supplement: Additional file 1: — Surveys - Rounds 1, 2 and 3. (PDF 275 kb) [file 12961_2017_252_MOESM1_ESM.pdf]

NIHR research priorities for health inequalities- Delphi survey

Introduction

Thank you for agreeing to take part in our survey on research priorities in health inequalities. (Just for clarity, we are using the WHO definition of health inequalities as "differences in health status or in the distribution of health determinants between different population groups").

We are interested in hearing about problems relating to health inequalities where evidence would help practice. The ultimate aim of this project is for us to fund research to fill important evidence gaps identified, and hence inform future practice. You may wish to discuss relevant issues with your colleagues, or possibly delegate to someone else within your organisation to respond on your behalf.

We are interested to hear about issues affecting you, these could be local issues where your organisation could implement an intervention, or the issues could be much wider affecting the whole UK. Unfortunately we are not able to progress with ideas on government policy or fiscal issues (e.g. tax changes, benefits, legal minimum age limits for alcohol consumption etc.) as this would be outside the remit of our research programmes.
http://www.nets.nihr.ac.uk/funding

1. Please check the box below to indicate that you have read and understood the information in the Participant Information Sheet. click here.

◯ I have read and understood the information in the Participant Information Sheet

Round 1 Question. Please give a maximum of 3 responses (responses to be given in free text). In your opinion what are the most important questions/problems in health inequalities in the UK where evidence would be helpful? What are your reasons for selecting this/these question(s)/problem(s)?

2. Suggestion 1 and reason

3. Suggestion 2 and reason

4. Suggestion 3 and reason

NIHR research priorities for health inequalities- Delphi survey

Your role and your organisation

Please could you give us some information about your role and your organisation?

5. What is your job title?

[ ]

6. What sort of organisation do you work for?

- [ ] Lower tier
- [ ] Upper tier
- [ ] Unitary

Other (please specify)

[ ]

7. Population served

- [ ] <100K
- [ ] 100-500K
- [ ] >500K

8. Geographical location of your organisation

- [ ] North of England
- [ ] Midlands and East of England
- [ ] London
- [ ] South of England
- [ ] Northern Ireland
- [ ] Scotland
- [ ] Wales

Thank you

Thank you for participating in this survey, your help is much appreciated.

We anticipate sending the questions for Round 2 in about one month and look forward to hearing your views.

On closing this survey you will be taken to our web site.

Delphi survey (Round 2)

Introduction

Dear Participants
Firstly, to those of you who gave us your views and suggestions about health inequalities in Round 1 of this Delphi survey, a big “thank you”. There were lots of thoughtful and varied ideas put forward. We out similar suggestions into groups – then ranked the groups by the number of suggestions they incorporated. In this second round we are asking you to further refine the top 3 topic priorities identified by the group. If you did not complete Round 1 we would still like to hear from you, so please do complete Round 2.

For each of the 3 priority topics please could now tell us which of the areas within the topic you consider to be the highest priorities requiring research evidence and the kinds of questions you need answers to in your professional capacity? You may wish to consider the dilemmas you face in your working role, or where to allocate resources in order to maximise the health of the population. Which of these areas most urgently needs research evidence?

1. Topic 1. Mental health

The priority which the highest numbers of respondents told us about was mental health. Specific areas mentioned by respondents included: prevention of serious mental health problems; building resilience and self-esteem; the mental health of specific groups including young people and school children, women, members of BME communities and older people; dementia; and the link between mental health and the social determinants of health.

Q1.In which areas of your practice related to mental health do you feel that you need new information/evidence?

Would your practice be likely to change if there was new evidence?

2. Topic 2. Environmental issues

The next highest priorities respondents told us about centred on environmental issues, some of which are inter-linked. Specific areas mentioned by respondents included: the impact of providing and maintaining good quality green and natural spaces and access to those spaces; the built environment including housing and areas of high deprivation; transport; and issues relating to rural communities.

Q2.In which areas of your practice related to environmental issues do you feel that you need new information/evidence?

Would your practice be likely to change if there was new evidence?

3. Topic 3. Health behaviours

The third set of priorities centred on health behaviours. Topics suggested included: smoking; alcohol consumption; inactivity; overweight and obesity, and healthy eating.

Q3. In which areas of your practice related to health behaviours do you feel that you need new information/evidence?

Would your practice be likely to change if there was new evidence?

4. Q4. Having seen these thoughts from colleagues do you have any other ideas on these areas or perhaps on unrelated topics?

Delphi survey (Round 2)

Your role and your organisation

Please could you give us some information about your role and your organisation?

5. What is your job title?

6. What sort of organisation do you work for?

- [ ] Lower tier
- [ ] Upper tier
- [ ] Unitary

Other (please specify)

7. Population served

- [ ] <100K
- [ ] 100-500K
- [ ] >500K

8. Geographical location of your organisation

- [ ] North of England
- [ ] Midlands and East of England
- [ ] London
- [ ] South of England
- [ ] Northern Ireland
- [ ] Scotland
- [ ] Wales

Delphi survey (Round 2)

Thank you

Thank you for participating in this survey, your help is much appreciated.

We anticipate sending the questions for Round 3 in about one month and look forward to hearing your views.

We will keep the ideas on other topic areas mentioned such as employment, health literacy, PPI etc. for possible future work. This is a pilot scheme for the identification of topics; we may run further surveys on other topics if it is successful.

Topics out of the remit of the NIHR Public Health research programme, such as topics relating to NHS services we will pass on the appropriate funding streams.

We are able to take forward topics relating to locally determined policy but not national policy, such as changes to the welfare system.

On closing this survey you will be taken to our web site.

National Institute for Health Research

Introduction Round 3

Dear Participants

A big “thank you” again for those of you who responded in Rounds 1 and 2.

In Round 2, within each of our main topic areas (mental health, environmental issues and health behaviours), you told us which areas of your practice would benefit most from new information/research evidence. Our aim in this next round is to further refine your ideas, and ask your opinions about how these issues may be tackled.

Another theme that came out very strongly was “Implementing evidence”. We will address that as a fourth topic area.

If you were unable to respond in Rounds 1 or 2, we would still very much like to have your views in this third round.

1. Topic 1. Mental health
The main themes which emerged in “Mental health” are summarised below:

1. Building resilience and self-esteem: There is a need to build resilience and self-esteem, both in individuals and communities to prevent mental health problems. There is greater need for this in particular groups such as minority ethnic groups and looked after children. Building resilience and self-esteem in children links to social determinants of health and could have long term benefits. Evidence on effective interventions to inform practice is lacking.
2. Prevention and early diagnosis: There is a need for prevention and early response for mental health problems, including community interventions outside of healthcare, both for common mental health problems and serious mental illness. Interventions to prevent suicide were also identified as important.
3. Social isolation: Social isolation is a problem, particularly among minority ethnic groups and older people.
4. Minority ethnic groups: Mental health in minority ethnic groups is of concern, particularly communities where mental health issues are stigmatised.

We would now like to get your thoughts about possible interventions (things which could be done) which might address the following problems which you have told us about.

For example: interventions in schools to promote resilience; or media campaigns to mitigate unwanted social isolation. These are just examples of the kinds of things that might be done, but we want your views on what you think is needed and where evidence might help you in your work.

If you have no interest or no expertise in this area please leave the questions blank.

Question 1. Which interventions (things that can be done) would you like to see researched and in which population groups would you like to see this research done?

Please enter all you suggestions into the box below numbering them: 1, 2, 3 etc

2. Topic 2. Environmental issues

The main themes which emerged in “Environmental issues” are summarised below:

1. Health impact of planning (including prevention and wellbeing): Evidence is lacking to support impact of design of housing, towns, estates and planning relating to health and wellbeing. Evidence is needed to show whether good design can draw people together to interact positively and promote wellbeing; provide an environment to support healthy weight; and enable older people to maintain their independence.
2. Green or open space: Evidence of impact of green/open space on health and wellbeing. Access to green space and inequality of access to green space.
3. Housing quality: Good quality evidence is needed on the effect of the quality of housing on health.
4. Areas of deprivation: More evidence is needed on effective strategies for reducing deprivation and social inequalities in the short, medium and long-term, and investigating effects on health of improving the living environment in deprived areas.
5. Active transport/transport: Evidence of what works (including return on investment/ value for money assessments) to reduce obesogenic environments and improve active travel and giving priority to public transport to inform PH commissioning priorities.
6. Food production and environment: Evidence is needed relating to the food environment and quality of nutrition and balanced diet for children.

We would now like to get your thoughts about possible interventions (things which could be done) which might address the following problems which you have told us about.

For example: interventions to promote access to green space; or to improve the quality of housing stock. These are just examples of the kinds of things that might be done, but we want your views on what you think is needed and where evidence might help you in your work.

If you have no interest or no expertise in this area please leave the questions blank.

Question 2. Which interventions (things that can be done) would you like to see researched and in which population groups would you like to see this research done?

Please enter all you suggestions into the box below numbering them: 1, 2, 3 etc

3. Topic 3. Health behaviours

The main themes which emerged in “Health behaviours” are summarised below:

1. Multiple health behaviours: Evidence on interventions to address multiple health behaviours and clustering of unhealthy behaviours is needed. Evidence is also needed to inform whole environment approach to tackling obesity.
2. Physical activity: Evidence is needed looking at physical activity in females; and most effective ways of overcoming work and lack of time as barriers to physical activity.
3. Children and young people: Evidence is needed for behaviour change interventions to improve the health of children and young people, including specific groups such as looked after children and those from deprived neighbourhoods.
4. Real world evidence: “Real world” evidence on behaviour change is needed, where the wider context is considered.

We would now like to get your thoughts about possible interventions (things which could be done) which might address the following problems which you have told us about.

For example: interventions in schools to promote activity in females; or location of fast food outlets. These are just examples of the kinds of things that might be done, but we want your views on what you think is needed and where evidence might help you in your work.

If you have no interest or no expertise in this area please leave the questions blank.

Question 3. Which interventions (things that can be done) would you like to see researched and in which population groups would you like to see this research done?

Please enter all you suggestions into the box below numbering them: 1, 2, 3 etc

4. Topic 4 Implementing evidence

“Implementing evidence” emerged as a strong theme.

We would now like to get your thoughts about what interventions (things could be done) would help you to implement evidence in your work?

If you have no interest or no expertise in this area please leave the questions blank.

Question 4. What interventions do you think are needed, what might help you in your work?

Your role and your organisation

If you have not already done so, please could you give us some information about your role and your organisation?

5. What is your job title?

[ ]

6. What sort of organisation do you work for?

- [ ] Lower tier
- [ ] Upper tier
- [ ] Unitary

Other (please specify)

[ ]

7. Population served

- [ ] <100K
- [ ] 100-500K
- [ ] >500K

8. Geographical location of your organisation

- [ ] North of England
- [ ] Midlands and East of England
- [ ] London
- [ ] South of England
- [ ] Northern Ireland
- [ ] Scotland
- [ ] Wales

NHS
National Institute for Health Research

Thank you

Thank you for participating in this survey, your help is much appreciated.

We will keep the ideas on other topic areas mentioned such as employment, health literacy, PPI etc. for possible future work. This is a pilot scheme for the identification of topics; we may run further

surveys on other topics if it is successful.

Topics out of the remit of the NIHR Public Health research programme, such as topics relating to NHS services we will pass on the appropriate funding streams.

We are able to take forward topics relating to locally determined policy but not national policy, such as changes to the welfare system.

Phase 2 Survey- Introduction

Dear Participants

As explained in our letter inviting you to participate in this study, The NIHR Public Health Research Programme is interested in advertising for research addressing health inequalities. Our participants in Phase 1 of this study have identified the following two "themes" as high priorities for research: "Community interventions for prevention of mental health problems" and "Food and alcohol environment". They have told us that there is a need for research evidence to inform practice.

We would like to draw on your expertise to help us frame tractable research questions for these two topics, and to alert us to relevant methodological issues which should be mentioned in the commissioning brief, (the call advertising for research).

1. Theme 1 identified in the first phase: "Community interventions for prevention of mental health problems".

This theme includes:

i. Research into effective interventions in out of school community settings, including involvement of families.
ii. Population led research to highlight awareness and garner understanding via narrative to break down barriers.
iii. The need to reduce social isolation and increase social capital

Please add your suggested tractable Research Question(s) for the theme:"Community interventions for prevention of mental health problems":

2. Please tell us about any methodological issues which should be mentioned in the commissioning brief on "Community interventions for prevention of mental health problems":

3. Theme 2 identified in the first phase: “Food and alcohol environment”.

This theme includes:

i. More evidence on different communities' attitudes to food
ii. Evidence into the effect of environmental interventions in terms of food choice
iii. Effectiveness of different approaches to improving the food environment
iv. Interventions to influence the 'drinkscape'

Please add your suggested tractable Research Question(s) for the theme "Food and alcohol environment":

4.
Please tell us about any methodological issues which should be mentioned in the commissioning brief on “Food and alcohol environment":

NHS
National Institute for Health Research

Your role and your organisation

Please could you give us some information about your role and your organisation?

5. What is your job title?

6. Name of your organisation/institution

7. Geographical location of your organisation/institution

- ☐ North of England
- ☐ Midlands and East of England
- ☐ London
- ☐ South of England
- ☐ Northern Ireland
- ☐ Scotland
- ☐ Wales

NHS National Institute for Health Research

Thank you

Thank you for participating in this survey, your help is much appreciated.

We are able to take forward topics relating to locally determined policy but not national policy, such as changes to the welfare system.
